# Patient selection and treatment response assessment in radium-223 therapy

**DOI:** 10.1186/1470-7330-15-S1-P38

**Published:** 2015-10-02

**Authors:** M Kay, F Sundram

**Affiliations:** 1 University Hospital Southampton, Tremona Road, Southampton, Hampshire, SO16 6YD, UK

## Learning objectives

Radium-223 is an alpha emitter, licensed in 2013 for treatment of symptomatic bone metastases in metastatic castration resistant prostate cancer. The treatment consists of 6 intravenous administrations and ongoing patient management before, during and after treatment. In our institution, this service is led by the nuclear medicine department

The ALSYMPCA trial, published in 2013, demonstrated improved overall survival (14.9 vs 11.3 months) and prolonged time to first symptomatic skeletal event (15.6 vs 9 months) for patients administered Radium-223 vs placebo

Imaging plays a key role in the selection criteria and response assessment for this new therapeutic modality. We present an educational review of the spectrum of multimodality imaging techniques employed for pre-therapeutic assessment and also for therapeutic response assessment

## Content organisation

• A brief overview of the criteria for treatment with radium-223 will be provided

• Examples of CT and MRI scans of patients suitable for treatment, including pre and post therapeutic findings

• Isotope bone scans of patients before and following treatment, demonstrating the effect of radium-223 on skeletal lesions.

## Conclusion

Radium-223 is an emerging innovative treatment for symptomatic bone metastases from castrate resistant prostate cancer and may well be the standard of care in the future. Cancer imagers should be aware of the suitability criteria for treatment, which will help inform discussions when patients are being considered for this treatment. They should also be aware of the multimodality imaging findings on post therapeutic response assessment studies.

